# Mechanistic Aspects of the Palladium‐Catalyzed Suzuki‐Miyaura Cross‐Coupling Reaction

**DOI:** 10.1002/chem.202101880

**Published:** 2021-08-09

**Authors:** Massimo C. D'Alterio, Èric Casals‐Cruañas, Nikolaos V. Tzouras, Giovanni Talarico, Steven P. Nolan, Albert Poater

**Affiliations:** ^1^ Institut de Química Computacional i Catàlisi Departament de Química Universitat de Girona c/ Maria Aurèlia Capmany 69 17003 Girona Catalonia Spain; ^2^ Dipartimento di Scienze Chimiche Università di Napoli Federico II Via Cintia I-80126 Napoli Italy; ^3^ Department of Chemistry and Center for Sustainable Chemistry Ghent University Building S3, Krijgslaan 281 9000 Gent Belgium

**Keywords:** C−C bond formation, Cross coupling, DFT, palladium, preactivation, Suzuki-Miyaura

## Abstract

The story of C−C bond formation includes several reactions, and surely Suzuki‐Miyaura is among the most outstanding ones. Herein, a brief historical overview of insights regarding the reaction mechanism is provided. In particular, the formation of the catalytically active species is probably the main concern, thus the preactivation is in competition with, or even assumes the role of the rate determining step (rds) of the overall reaction. Computational chemistry is key in identifying the rds and thus leading to milder conditions on an experimental level by means of predictive catalysis.

## Graphical abstract

**The complexity** of the Pd‐catalyzed Suzuki‐Miyaura C−C bond formation reaction lies in the indivdual steps of the cycle, as well as in the generation of the catalytically active species. An overview of the topic is provided from a historical perspective.

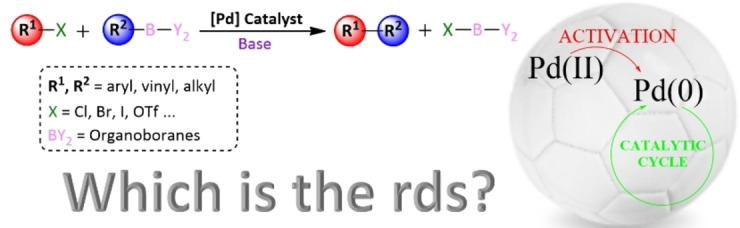

## 1 Introduction

### 1.1 Cross‐coupling reactions

Carbon‐carbon bond formation reactions are essential for the development of bioactive molecules, agrochemicals and drugs. In addition, they are also used to construct new organic materials with innovative electronic, optical or mechanical properties in the field of nanotechnology.^[1]^ In fact, these types of reactions, known as cross‐coupling reactions, have been some of the greatest breakthroughs in the fields of organic/organometallic synthesis since the discovery of catalysis.^[2]^ That is why over the last half century, C−C bond formation reactions have been extensively studied and new methodologies have been developed using transition metals to mediate reactions in a controlled and selective manner, under mild conditions. Of note, many of the transformations in question would not be possible to achieve without transition metal‐based catalysts. The paramount contribution of these reactions to chemistry was recognized with the award of the Nobel Prize in Chemistry in 2010 to three of their main developers, Richard F. Heck, Ei‐ichi Negishi and Akira Suzuki.^[3]^

**Scheme 1:**
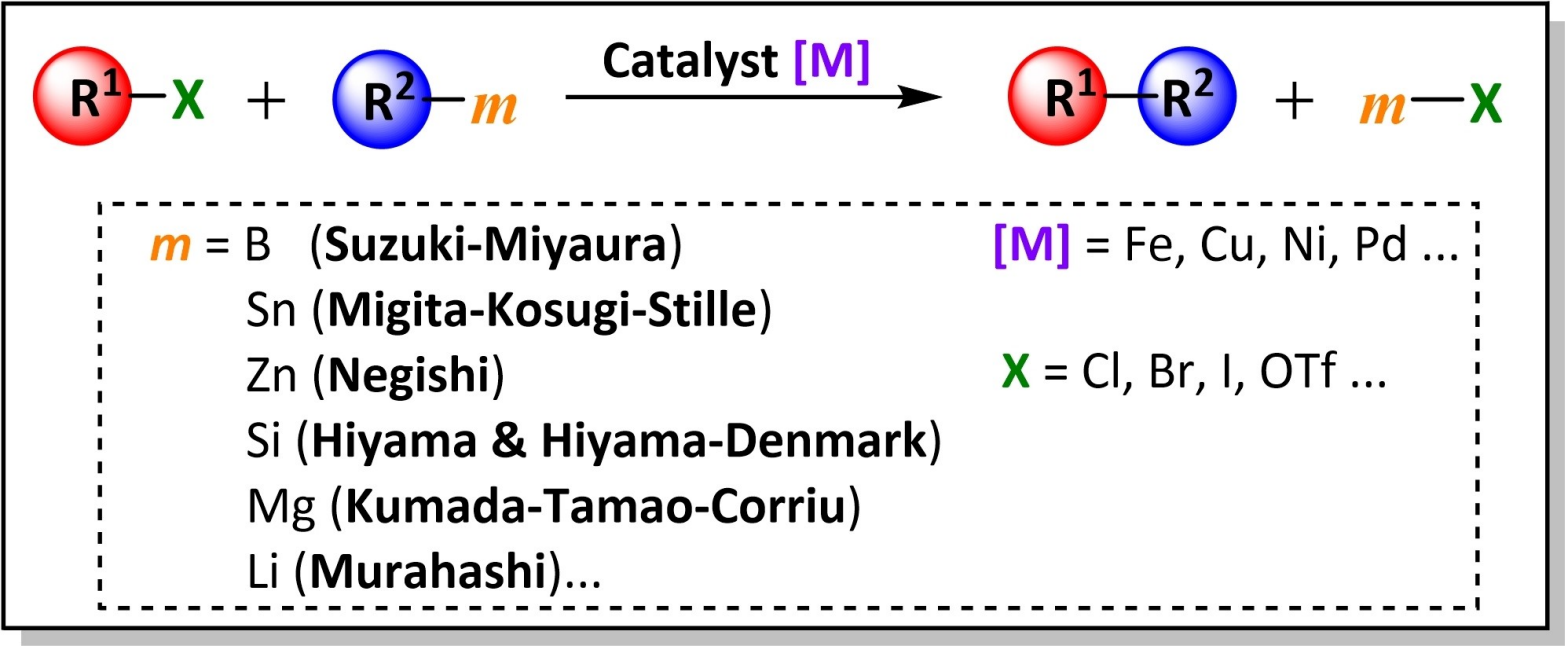
General scheme for C−C cross‐coupling reactions.

C−C cross‐coupling reactions are defined as carbon‐carbon bond formation reactions between an organic electrophile (*R^1^‐X*) and an organometallic nucleophile (*R^2^‐m*) in the presence of a metal catalyst [*M*] (Scheme 1). Commonly, the catalysts used in these reactions, are transition metal complexes of groups 8–10, in particular nickel and palladium. The main reason why these two metals are involved in the vast majority of cross‐coupling reactions is related to their ease of exchange of redox states, Ni(II)/Ni(0) and Pd(II)/Pd(0), which is an essential requirement to complete the catalytic cycle. The C−C cross‐coupling reactions are frequently sorted by the metal present in the nucleophile. For example, the Negishi reaction utilizes organozinc reagents, the Stille reaction utilizes organotin reagents, the Suzuki‐Miyaura reaction utilizes organoboron reagents, etc.

### 1.2 Origins of the cross‐coupling reactions

**Scheme 2:**
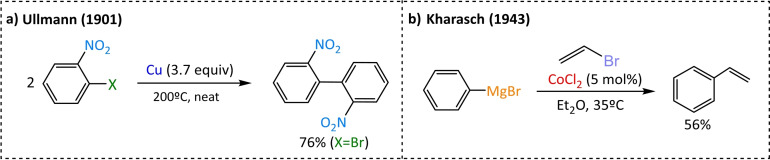
Ullmann and Kharasch C−C bond formation achievements.

We go back to the origin of cross‐coupling reactions and how they have ushered in being selective catalytic methods to construct C−C bonds, whether they are *sp*, *sp* ^*2*^ or *sp* ^*3*^ carbon centres, in order to obtain functionalized compounds. The first C−C cross‐coupling as its proper definition implies dates back to 1943 when Kharasch's work reached the first cross‐coupling between a vinyl bromide and an arylmagnesium species using cobalt chloride (Scheme 2), although the use of metals to facilitate organic transformations started in the beginning of the 20^th^ century.[^4^, ^5^]

In the early 1900s, Ullman (1901) achieved the formation of C−C bonds by the homocoupling of 2‐bromonitrobenzene, using Cu as a metal and without the presence of organometallic reagents (Scheme 2).^[6]^ Later, in 1923 Job reported that phenyl‐MgBr, in the presence of NiCl_2_, was able to absorb CO, NO, C_2_H_4_, C_2_H_2_ and H_2_; thus, this was the first time that a metal was efficiently combined with organometallic reagents (a Grignard reagent).

Despite using the transition metal in catalytic amounts for cross‐coupling reactions after the Kharasch discovery, these reactions were not as selective as they are currently. This trend continued until the middle of the century when Cadiot‐Chodkiewicz (1955) and Castro‐Stephens (1963) achieved for the first time a selective cross‐coupling between *sp* and *sp* carbon centres and *sp* and *sp* ^*2*^ carbon centres, respectively. From 1963 until now, C−C cross‐coupling reactions have undergone several advances, considering that the second half of the 20^th^ century is when the most outstanding C−C bond formation reactions have been discovered.

**Scheme 3:**
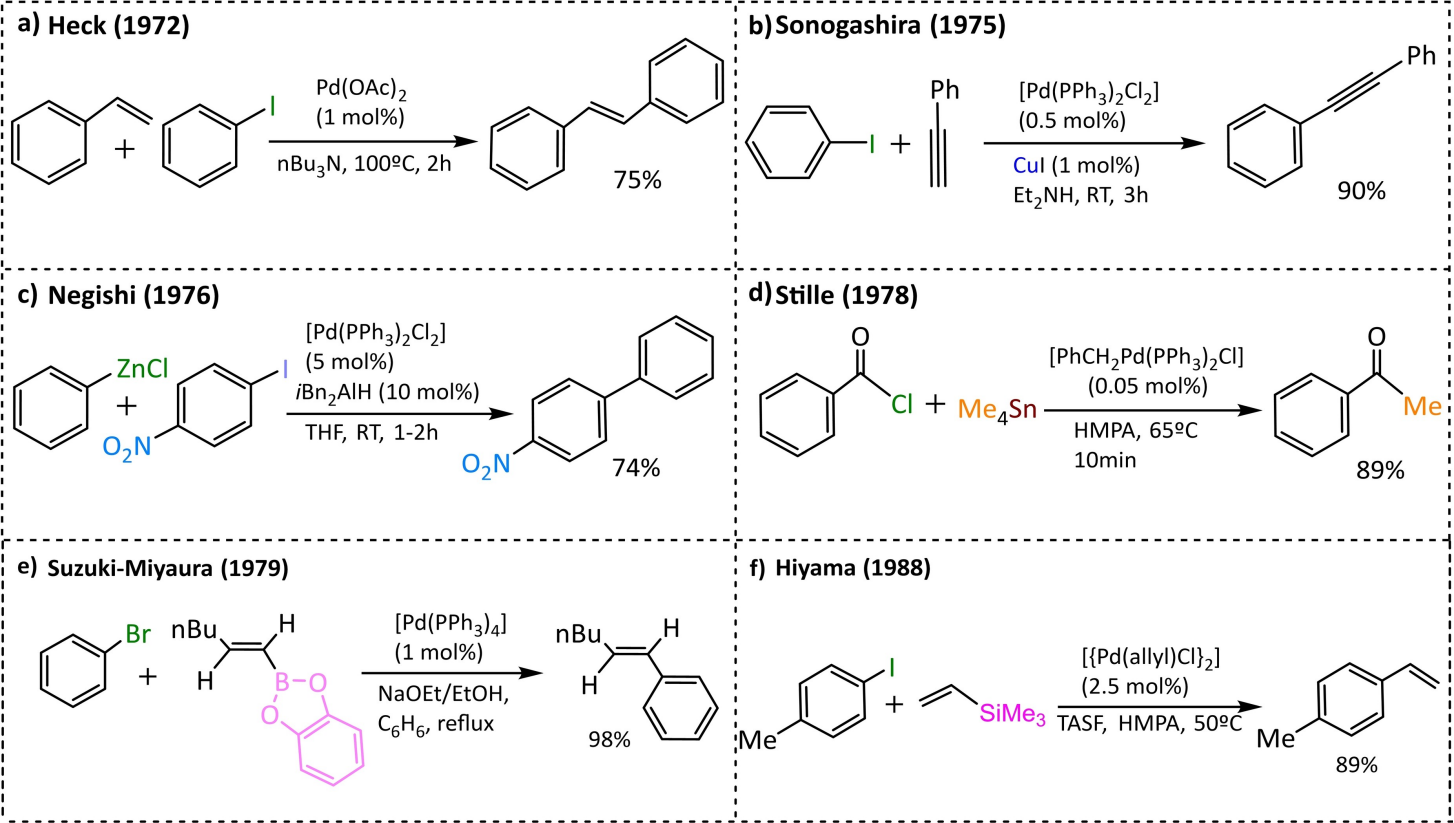
Highlighted C−C cross‐coupling reactions, catalysed by Pd, presented in chronological order of discovery.

The pioneering study of Walter Hafner on palladium‐based catalysts for the production of ethylene oxide from ethylene paved the way for the use of palladium in cross‐coupling reactions. Indeed, for the first time a palladium π‐allyl complex was characterized.^[7]^ Based on these studies on palladium, Mizoroki (1971) and Heck (1972) discovered the Heck reaction (also known as Mizoroki‐Heck reaction). These two scientists achieved the first coupling between an aryl halide and an alkene using a palladium catalyst (Scheme 3a).[^8^, ^9^, ^10^, ^11^] Later on, Corriu (1972) and Kumada (1972) independently carried out a cross‐coupling catalysed by nickel, between an aryl and an alkenyl halide with Grignard reagents.[^12^, ^13^, ^14^, ^15^] Furthermore, it must be highlighted that Kumada introduced the use of phosphine ligands to modulate the reactivity of the metal. However, in 1975 Murahashi substituted nickel with palladium because it increased the stereoselectivity,[^16^, ^17^] broadened the scope of Kumada's reaction and enhanced the reactivity of the metal by modulating the ligands. Simultaneously, Sonogashira improved the coupling between *sp* and *sp* ^*2*^ carbon centres by adding a copper co‐catalyst, using catalytic amounts of the transition metal as well as carrying out the reaction at room temperature (Scheme 3b).^[18]^ Of note, at least the Heck and Sonogashira reactions discussed here are not strictly speaking cross‐coupling reactions, since they involve the activation of a C−H bond instead of a C−Metal bond.

However, it was not until 1976 that other organometallic reagents were used as coupling partners. Substantial impact in this field was made by Negishi and co‐workers,^[19]^ who assembled an aryl halide with different organometallic reagents based on zinc, boron or tin. Nonetheless, Negishi focused his research in the cross‐coupling catalysed by palladium between the aryl halide and organozinc reagents (Scheme 3c). Following Negishi's research of using different organometallic nucleophiles, Stille achieved a palladium‐catalysed cross‐coupling of an organotin reagent with an aryl chloride (Scheme 3d),[^20^, ^21^] pointing out that the first example of Stille coupling presented is acyl coupling.[^22^, ^23^] The main drawback of this reaction is the toxicity of the organotin reagents.

Another class of organometallic reagents that Negishi studied was the organoborane nucleophiles. However, it would be Suzuki and Miyaura^[24]^ who in 1979 would lead the organoborane cross‐coupling reactions to the next level by coupling an alkenylborane with an aryl halide (Scheme 3e). In addition, the Suzuki‐Miyaura reaction can be performed under mild conditions,^[25]^ even though it requires the presence of a base. Although at the time it seemed that everything in cross‐coupling was discovered, the use of organosilicates as nucleophiles remained unexplored. In 1994 Hiyama described a nickel and palladium catalysed cross‐coupling of organosilanes with aryl halides,^[26]^ which had to be activated by the tris(dimethylamino)sulfonium difluorotrimethylsilicate (TASF) reagent (Scheme 3f).

**Scheme 4:**
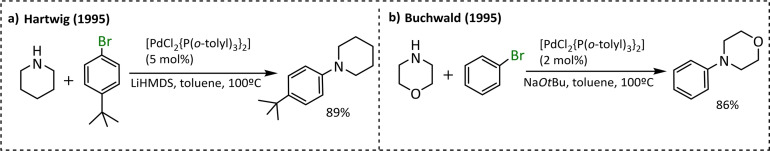
Highlighted C‐Het cross‐coupling reactions catalysed with Pd presented in chronological order of discoveries.

**Figure 1:**
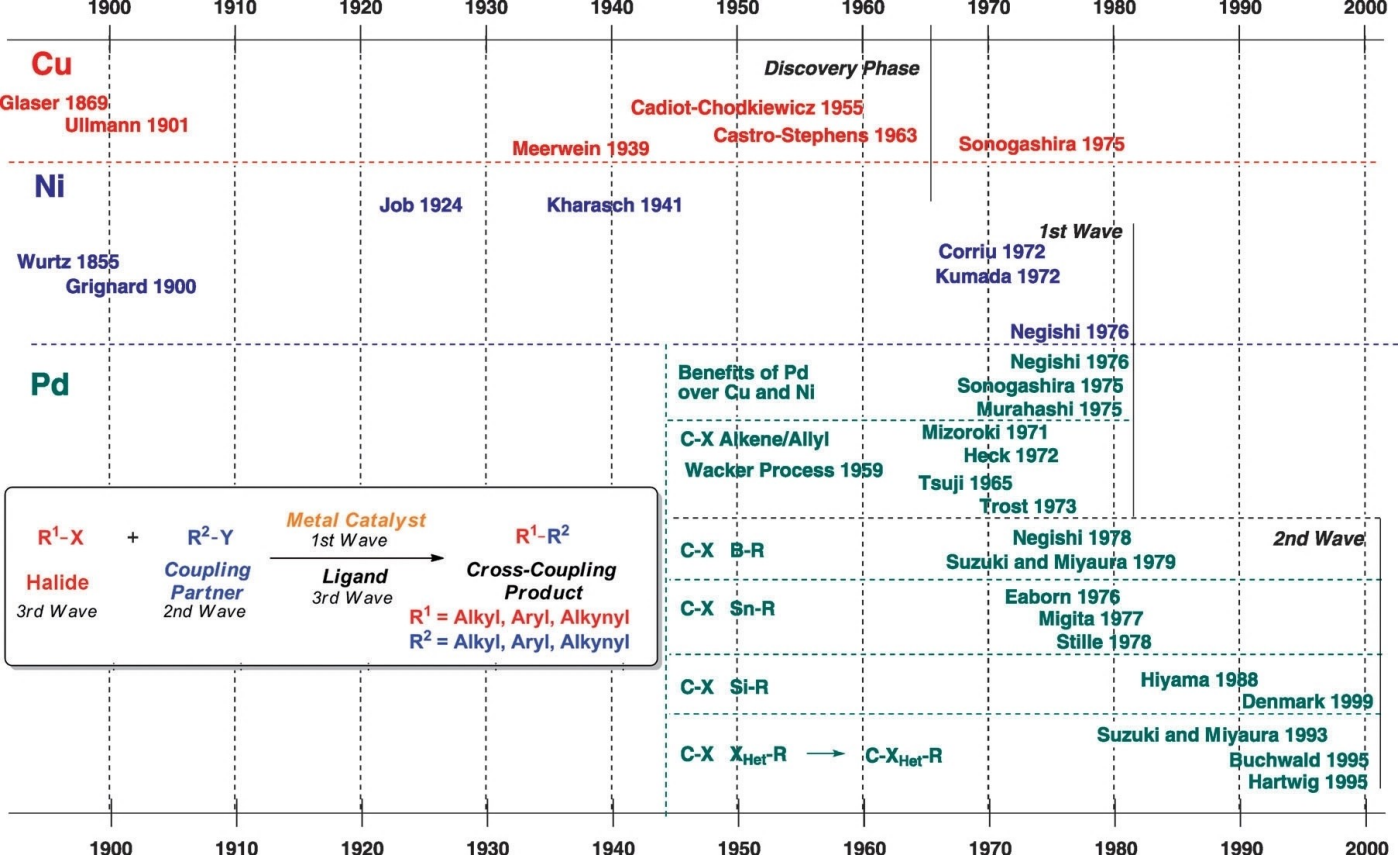
Timeline of the discovery and development of metal‐catalysed cross‐coupling reactions. Figure extracted from reference [4].

Another breakthrough in the world of cross‐coupling would be achieved when Hartwig (1995) and Buchwald (1995) achieved a cross‐coupling leading to C−N bonds, catalysed by palladium (Scheme 4).[^27^, ^28^] Since the formation of C−N bonds by the Buchwald‐Hartwig reaction, C‐Het coupling has been expanded to include C−O and C−S bonds among other heteroatoms by means of cross‐coupling reactions.^[29]^ The overall breakthroughs^4^ of the C−C cross‐coupling as well as the beginning of the C‐Het coupling are represented in the form of a timeline in Figure 1.

### 1.3 The Suzuki‐Miyaura cross‐coupling reaction

**Figure 2:**
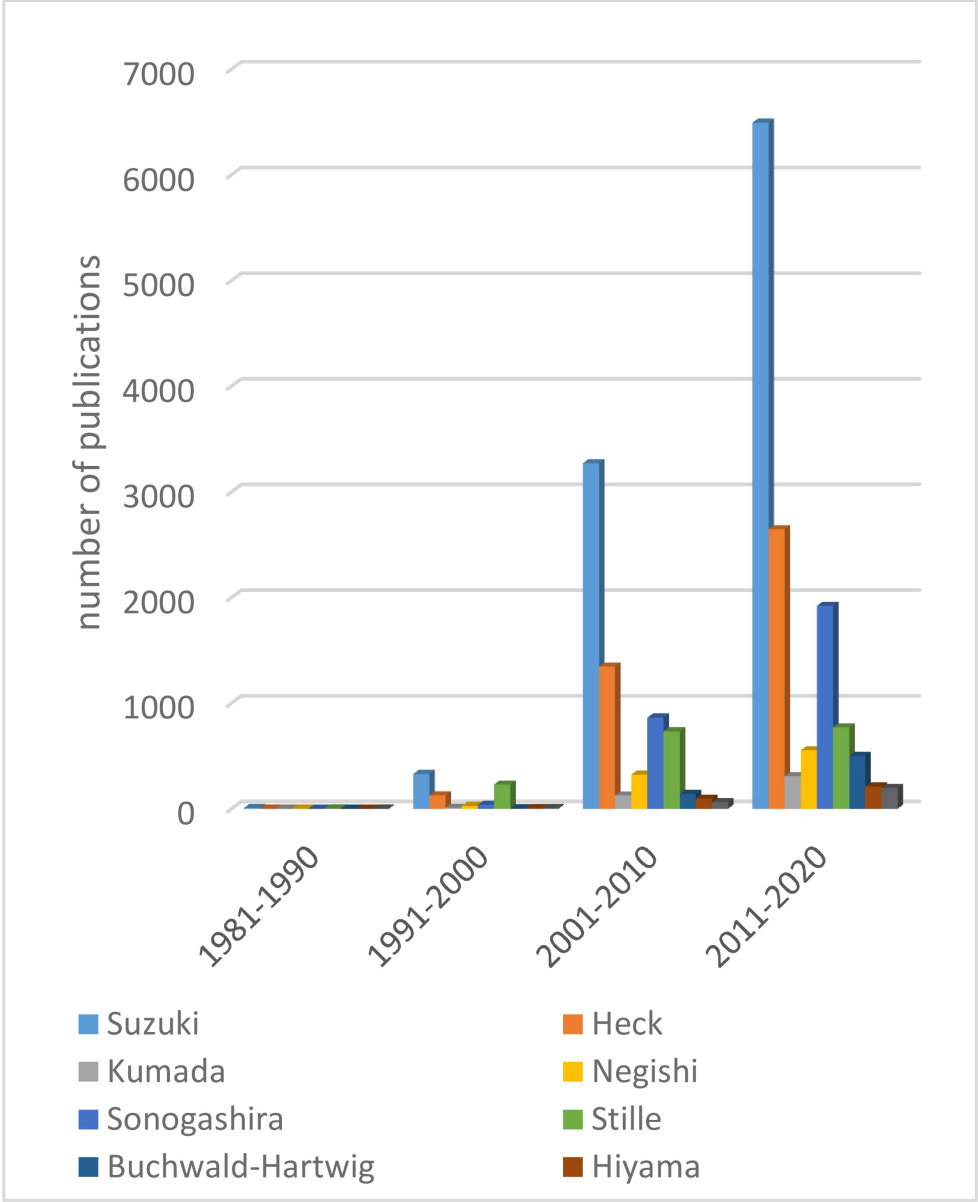
Increase of the cross‐coupling reports over the decades leading up to 2020 (source: Web of Science).

Among the cross‐coupling reactions, the Suzuki‐Miyaura (SM) reaction is the one that has witnessed the most noticeable growth since its discovery in 1979,^[30]^ as well as being one of the most widely used reactions to obtain biaryls and functionalized aromatic molecules (Figure 2).

**Scheme 5:**
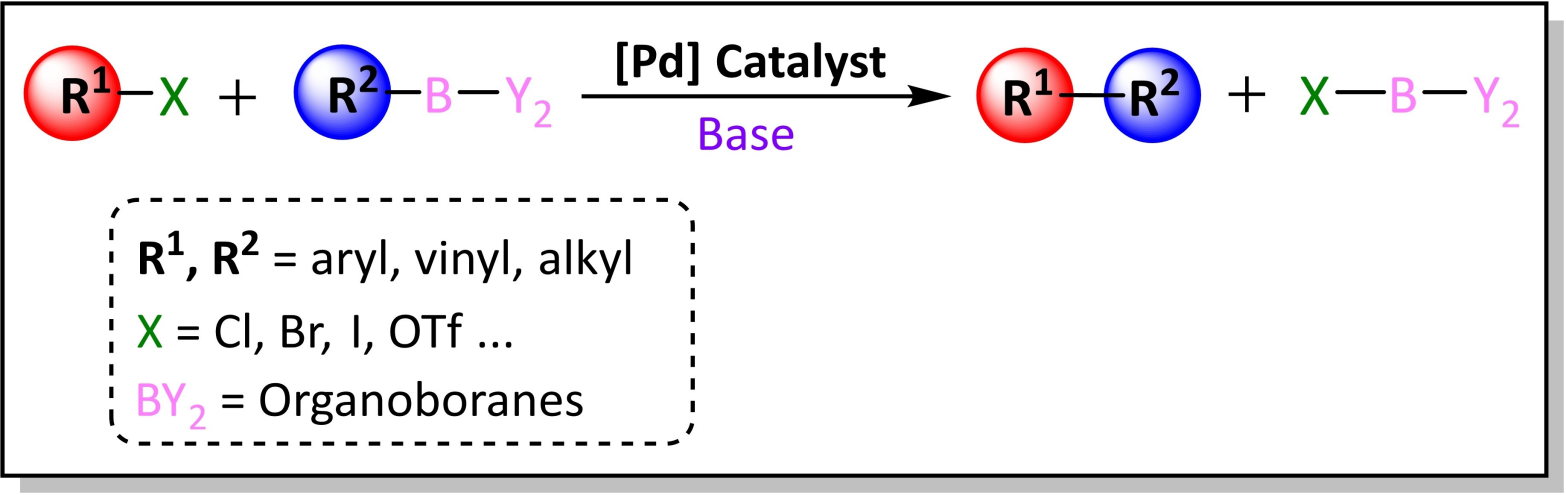
General scheme for the palladium catalysed Suzuki‐Miyaura cross‐coupling reaction.

The Suzuki‐Miyaura coupling is the cross‐coupling reaction that provides a general methodology for forming C−C bonds using various organoboron compounds (R^2^‐BY_2_) with organic electrophiles (R^1^‐X), for example halides or pseudohalides, catalysed by a palladium complex in the presence of a base (Scheme 5).

The SM reaction presents several advantages^[31]^ that stem from the environmentally benign nature of the organoboron reagents,[^32^, ^33^] which are readily available, less toxic, and air‐ and moisture‐stable. Indeed, the inorganic boron‐based by‐product can be easily removed after the reaction. In addition, they can react under mild conditions as well as under aqueous solvent and heterogeneous conditions.^[34]^ The reaction is only slightly affected by steric hindrance and has a wide tolerance for a broad range of functional groups.^[35]^ More importantly, the coupling proceeds with high regio‐ and stereoselectivity. Even if the catalyst is very expensive, the required amount is very small and it can be used in one‐pot strategies. The main drawback of this reaction is that a base is always necessary.

### 1.4 Palladium catalysts

Since the Suzuki‐Miyaura cross‐coupling reaction was discovered in 1979, many studies have been conducted, finding that the combination of the base, the solvent and the catalyst can affect the yields and selectivity of the Suzuki‐Miyaura reaction.^[36]^ The last topic is the one that has received more attention in recent years, as some research efforts have focused on developing novel catalysts based on first row transition metals, such as Fe, Cu, Ni, etc., due to their wider availability and large terrestrial abundance compared to those of Pd.^[37]^ Nonetheless, palladium based catalysts remain the optimal choice in the majority of the cross‐coupling reactions, even though more recently nickel catalysts have become a hot topic in catalysis due to of some advantages in certain reactions with respect to Pd,[^38^, ^39^, ^40^, ^41^, ^42^] apart from the obvious lower economic cost.[^43^, ^44^]

**Scheme 6:**
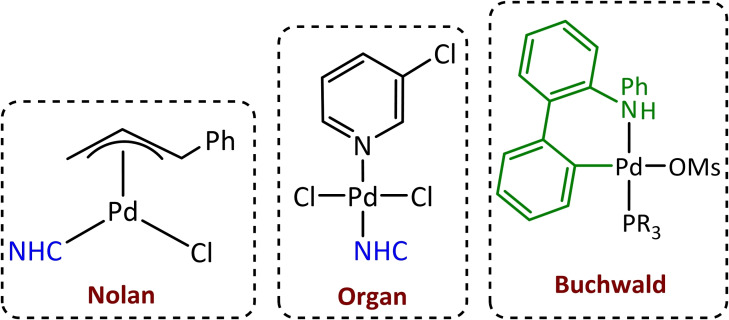
Types of pre‐catalysts bearing novel, electron‐donating and bulky phosphine and NHC ligands.

Initially, the palladium‐based catalysts used in cross‐coupling reactions employed simple phosphines, such as PPh_3_, as ligands. However, the need to overcome new challenging substrate transformations with higher selectivity and/or under mild conditions^[45]^ has caused the emergence of the ligand design field. Over the past 25 years, a better understanding of the steric[^46^, ^47^, ^48^, ^49^] and electronic^[46]^ properties of ligands[^50^, ^51^, ^52^] as well as the binding properties of metals has led to the creation of well‐defined organometallic complexes as highly effective (pre)catalysts.^[53]^ Therefore, the greatest success in the field of catalysis has been achieved through ligand design. The new phosphine‐type ligands^[54]^ used in palladium catalysis are strongly electrodonating and sterically bulky like tertiary phosphines (PR_3_) or the novel biaryl phosphine ligands developed mainly by Buchwald's group. At the same time, the Herrmann, Glorius, Organ and Nolan groups, carried out the development of N‐heterocyclic carbenes (NHCs) as ancillary ligands in cross‐coupling reactions (Scheme 6).[^55^, ^56^, ^57^, ^58^, ^59^, ^60^, ^61^, ^62^, ^63^] Contrary to the initially used tertiary and monodentate biaryl phosphines, NHCs had several features such as air, moisture, thermal and oxidative stability when coordinated, or better electronic properties[^64^, ^65^, ^66^] (strong σ‐donor ability) and distinct steric profiles, characteristics which encouraged their use.

Thus, these ancillary ligands contribute to the catalytic process owing to their electronic and steric properties by facilitating the oxidative addition of less reactive aryl halides,^[67]^ and increasing the rate of the reductive elimination. Additives such as olefins with electron‐withdrawing substituents were demonstrated to promote the activation of the catalyst.^[68]^ In order to catalyse the reaction, the palladium catalyst must be in its active form, which is a Pd^0^ complex. Recent studies show that monocoordinated species, [LPd^0^], tend to be favoured as active species versus dicoordinated complexes, [L_2_Pd^0^], and palladium(II) complexes, [L_2_Pd^II^] or [L_4_Pd^II^], because they do not need to be reduced or lose a ligand (L) through a dissociative process.[^69^, ^70^] Related to Pd(II)‐NHC hydroxide dimers, in analogy to amides, esters have also emerged as attractive acyl electrophiles,[^71^, ^72^] particularly interesting in the catalyst activation in the Suzuki cross‐coupling under aqueous conditions.

### 1.5 Generation of the active palladium species: well‐defined precatalysts vs. in situ formation

**Scheme 7:**
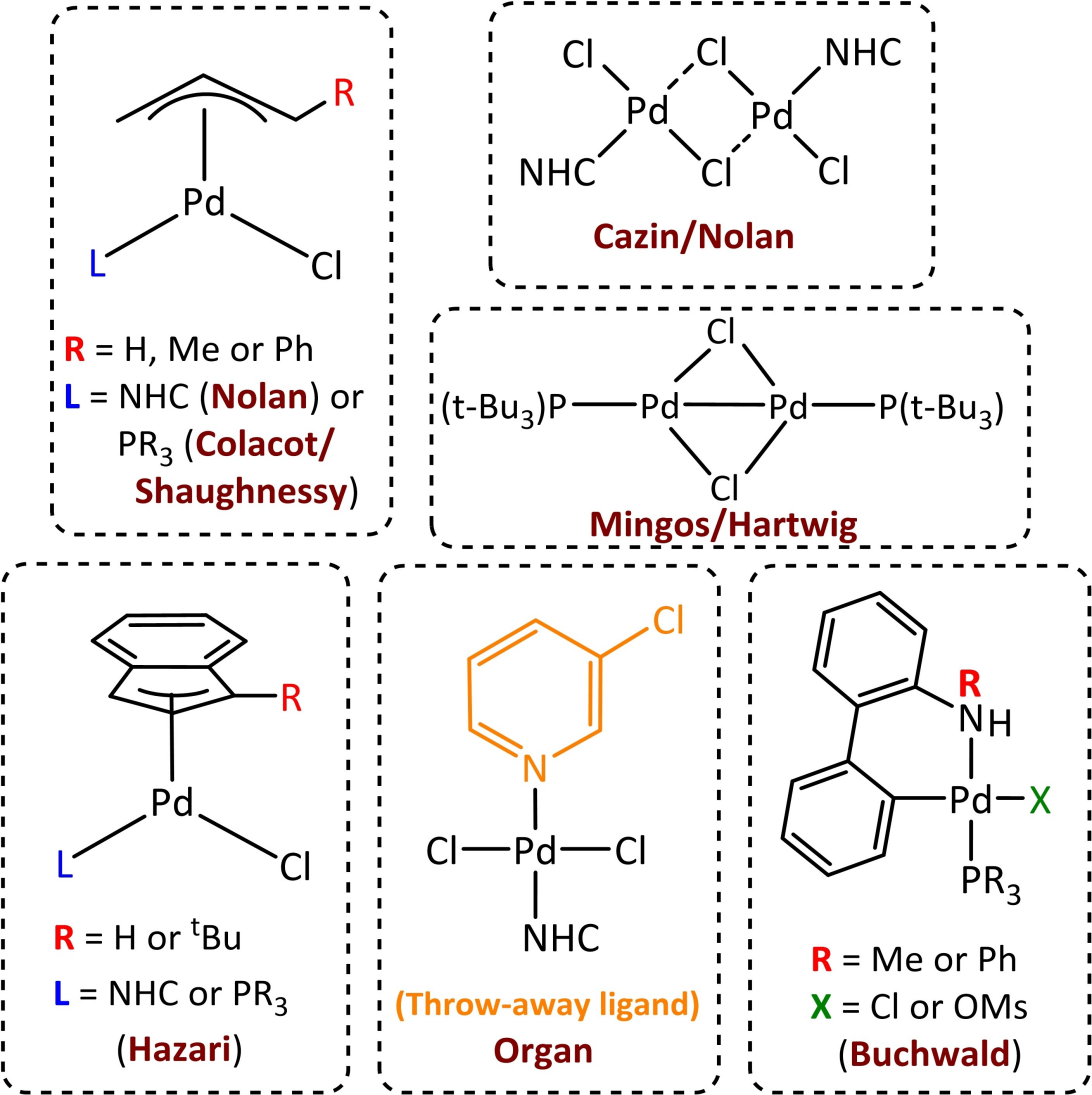
The main, well‐defined Pd precatalysts with a 1 : 1 Pd to ligand ratio.

As it is well known, the way the active palladium species L_n_Pd^0^ is generated affects the entire catalytic reaction. Traditionally, active species were obtained by mixing in situ an excess of ligand with the Pd^0^ source. However, obtaining the initial catalytic species by this method is not always optimal, since sometimes the precursor is cheaper than the novel ligands used and it has several drawbacks that have been documented in the literature. Thus, the way to create the active palladium species, L_n_Pd^0^, using tertiary phosphines or other bulky ligands can be divided into two groups, depending on the nature of the proposed catalytically active Pd^0^ species.^[73]^ L_2_Pd^0^ active species^[74]^ are highly active that can be formed by both palladium(0)^[75]^ and palladium(II) complexes, such as L_4_Pd^0^ and L_2_PdX_2_, and their reactivity is reflected in their air sensitivity in the solution phase. L_4_Pd^0^ complexes, such as Pd(PPh_3_)_4_ or Pd[P(t‐Bu)_3_]_2_, are air‐ and moisture sensitive which makes it challenging to obtain the catalytic species from a synthetic point of view. While, compared to the L_4_Pd^0^, L_2_PdX_2_ complexes are stable to air and moisture even at high temperatures in the solution phase in addition to being monodentate or bidentate. Therefore, the active species L_2_Pd^0^ is obtained through a ligand dissociation process if it is an L_4_Pd^0^ whereas if it is an L_2_PdX_2_ compound it must be reduced from Pd^II^ to Pd^0^ via *reductive elimination*. Until recently, L_2_Pd^0^ was also assumed to be the active species in a cross‐coupling catalytic cycle, although a better understanding of the effects of the catalyst and ligand, as well as the metal to ligand ratio has led to even more active catalysts, LPd^0^. This is logical, since the 12‐electron LPd^0^ complex is kinetically more active than the 14‐electron L_2_Pd^0^ species that are thermodynamically stable.^[76]^ In addition, computational and experimental studies support that the catalyst carrying sterically demanding ligands^[77]^ favoured the monocoordinated palladium active species.^[78]^ This new LPd^0^ species cannot be generated in situ because in solution more than one ligand would coordinate the Pd. Therefore, a precatalyst must be used to produce the active species.^[79]^ In detail, three main precursors exist, that achieve the 1 : 1 ratio: Pd^I^ dimers, Pd^II^(R‐allyl)Cl complexes and Pd^II^ palladacycles (Scheme 7). In addition, [Pd^II^(μ‐Cl)Cl(NHC)]_2_ complexes have been used as precatalysts for room temperature activation of aryl chlorides.^[80]^ Each precatalyst has its own pathway to produce the active species.^[81]^

Of the well‐defined precatalysts, the dimeric is the only one in which palladium oxidation state is +1. Major developments in the field of dimeric palladium precatalysts can be attributed to Schoenebeck and co‐workers,[^82^, ^83^, ^84^, ^85^] who have established that the formation of the Pd^I^ dimer depends on the conditions under which the reaction occurs even though the active species remains the Pd^0^ complex. Moreover, Schoenebeck's group demonstrated using computational tools, that the way to obtain the monodentate complex is done via *a reductive pathway* and not through the disproportionation mechanism that was assumed.^[86]^ On the other hand, the other types of precatalysts are Pd^II^ complexes. Palladacycles are air‐ and moisture stable precatalysts that have been used in several cross‐coupling reactions. Buchwald and co‐workers have created several generations of these precatalysts that can generate the active species by reacting with a base.[^87^, ^88^] Furthermore, the latest generation of palladacycles retains all the features of previous generations, such as bulky ligands and a mesylate anion even though they change the typical −NH_2_ group to −NHMe/Ph to avoid generating the carbazole by‐product that is suspected to be carcinogenic.

**Scheme 8:**
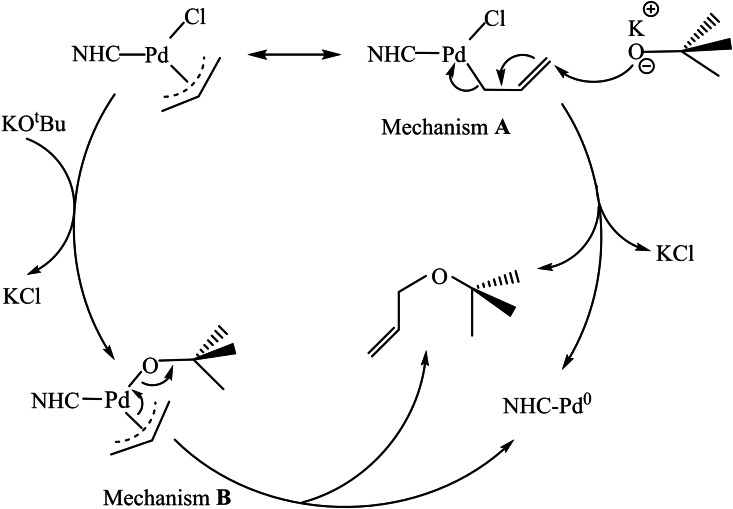
Two different mechanisms of activation for Nolan's π‐allyl precatalysts.^[94]^

Last but not least, Organ's PEPPSI[^89^, ^90^] and Nolan's π‐allyl precatalysts^[91]^ containing an NHC ligand have proven to be robust, air‐ and moisture stable and efficient in cross‐coupling reactions.^[92]^ The first precatalyst undergoes two consecutives transmetalations by an organometallic reagent, followed by a reductive elimination and the dissociation of the pyridine (throwaway ligand) to generate the active species. Nolan's allyl‐type precatalysts can generate the LPd^0^ species through two different mechanisms, one involving a single step by a nucleophilic intermolecular attack of the alcohol solvent and the other via *two steps*: a ligand substitution and a nucleophilic intramolecular attack by the alcohol solvent (Scheme 8).^[93]^

**Figure 3:**
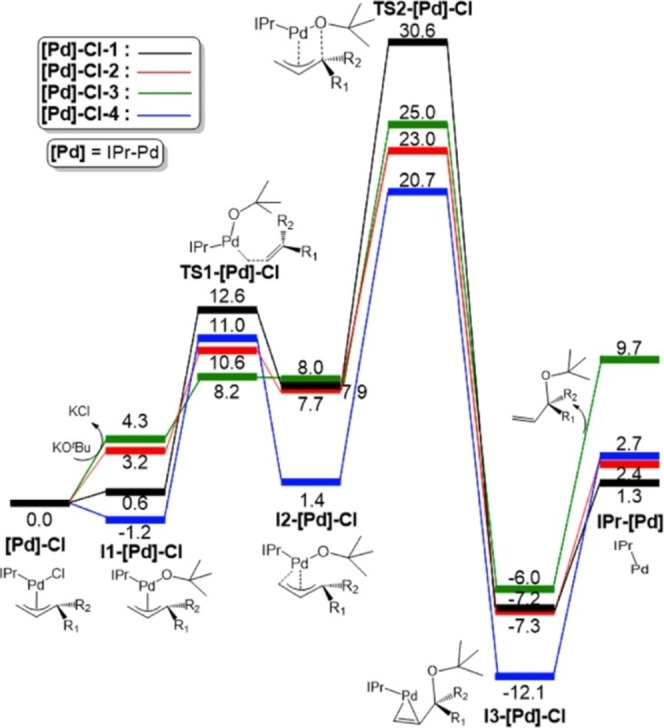
Computed stationary points of the activation mechanism for the studied precatalyst [Pd]‐Cl‐1−4 series ([Pd]‐Cl‐4, R_1_=R_2_=H; [Pd]‐Cl‐2, R_1_=H, R_2_=Me; [Pd]‐Cl‐3, R_1_=R_2_=Me; [Pd]‐Cl‐4, R_1_=H, R_2_=Ph). Relative Gibbs energies are given in kcal/mol. Figure extracted from Ref. [94].

At first glance, the mechanism followed for the activation of the catalyst may seem secondary. However, Poater and coworkers^[94]^ have demonstrated that the most energetically demanding step/reaction of the Suzuki‐Miyaura cross‐coupling catalysis, for this type of catalysts, does not belong to any step of the catalytic cycle, or its rate determining step (rds) but to a step of the precatalyst activation. Thus, here the induction period is crucial. On the other hand, the existence of the Pd(0) species is still under debate. In 2013 Vidossich, Ujaque and Lledós addressed the issue of solvent coordination in the Pd‐PPh_3_ system,^[95]^ showing the accessibility of species such as the bare monophosphine Pd(PPh_3_) species, often depicted in catalytic cycles, is actually not applicable in solution. In particular, by means of DFT calculations, Poater and coworkers unveiled the activation mechanism (Figure 3) for the precatalyst series [Pd]‐Cl‐1‐4 derived from [Pd(NHC)(R‐allyl)Cl] species with several terminal substituents on the allyl moiety.^[94]^ Calculations reproduce quantitatively the experimental trend of the activation mechanism for the [Pd]‐Cl‐1‐4 series. In particular, the lowest yield (12 %) displayed by [Pd]‐Cl‐1 species (ΔG^≠^≈30.0 kcal/mol) and of the high yields (≈90 %) observed in the case of [Pd]‐Cl‐2‐4 complexes (ΔG^≠^≈20.0 kcal/mol). The activation barriers are referred to the transition state leading to the formation of the active catalytic species NHC−Pd(0) occurring via reductive elimination, named as TS2‐[Pd]‐Cl in Figure 3.

Recently Hazari and co‐workers[^82^, ^96^] have improved the activation of the precatalyst by changing the allyl scaffold,^[97]^ instead of using a η^3^‐cinnamyl group they employ a η^3^‐1‐^t^Bu‐indenyl moiety that prevents the formation of the Pd^I^ dimer observed when the η^3^‐cinnamyl precatalyst is used. Furthermore, based on Nolan's precatalyst, the groups of Colacot and Shaughnessy have developed a precatalyst[^98^, ^99^] using Buchwald‐type phosphines ligands instead of NHC ligands.

Of note, there are explanations of why the activation is so complex, dedicating the efforts to rationalize dimeric species,^[100]^ which are often considered off‐cycle.[^101^, ^102^]

### 1.6 Catalytic cycle

**Scheme 9:**
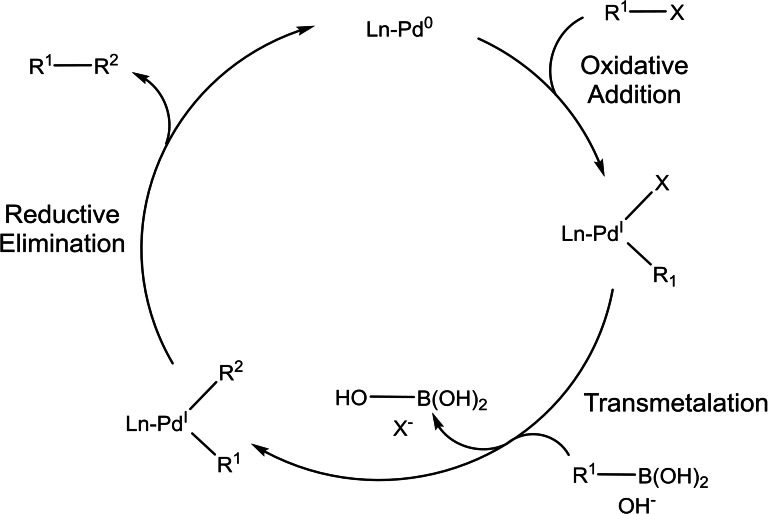
General catalytic cycle for Pd‐catalysed Suzuki‐Miyaura C−C cross‐coupling reaction.

Suzuki‐Miyaura cross‐coupling reactions follow a well‐defined catalytic cycle based on three steps^[103]^ (Scheme 9): *i) oxidative addition* of the organic halide or other electrophiles to the palladium(0) complex to give R^1^‐Pd^II^‐X; *ii) transmetalation* between the R^1^‐Pd^II^‐X and the boronate (R^2^‐BY_2_) with the assistance of a base; and finally, *iii) reductive elimination* to form a new C−C bond (R^1^‐R^2^) with the simultaneous regeneration of the catalyst. Among these steps, the first and last are common to all the cross‐coupling reactions and have been studied in depth by experimental and computational methods, which is why they are well understood. However, the transmetalation step is different in cross‐coupling reactions as they differ in the nucleophile used as well as the reaction conditions. Moreover, the experimental data in this step are pretty difficult to obtain due to the difficulty in isolating/characterizing key intermediates. Hence, it is not strange to know so little about the transmetalation step, as it is the least studied. As already mentioned, although it is not part of the three basic steps of a cross‐coupling catalytic cycle, the pre‐activation of the active species is an important step that will affect the overall reaction. Thus, the choice of a good precatalyst containing bulky and electron‐donating ligands will have a positive effect on the catalytic cycle, as it will enhance the oxidative addition and reductive elimination, as well as avoid the problem of an undesired side reaction, a β*‐elimination*.

### 1.7 Oxidative addition

**Scheme 10:**
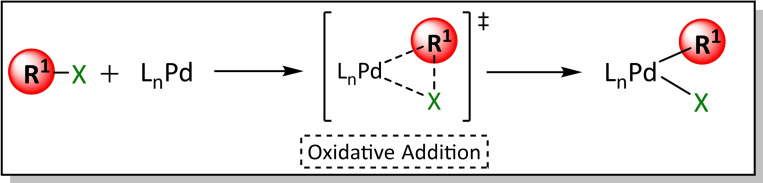
General equation for the oxidative addition step.

The first step in the catalytic cycle is the oxidative addition in which the bond between the heteroatom X and the organic R^1^ group is broken, and two new bonds are formed with the LPd^0^ metal complex, oxidizing the metal. Specifically, the formation of these bonds increases the oxidation state of the metal by two units. In addition, the ease of oxidative addition (OA) is known to be influenced by the strength of the C−X bond; thus, this step is dependent on the ligand of the Pd^0^ active species that is formed prior to the OA step (Scheme 10).

**Scheme 11:**
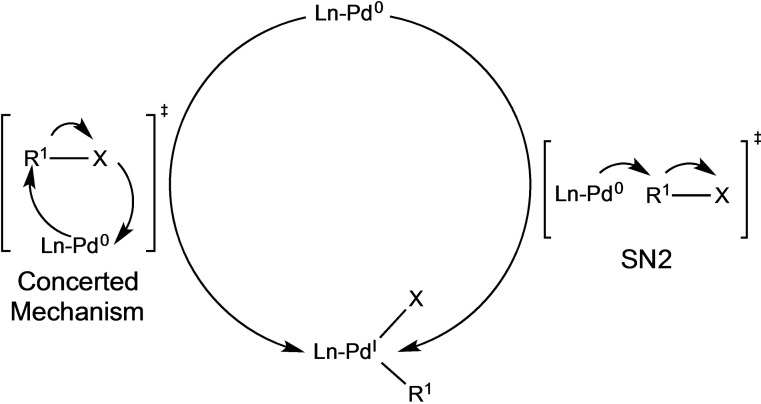
Plausible pathways of the oxidative addition step.

In 2013 Maseras et al.^[104]^ studied the mono‐ and dicoordinated palladium complexes in the oxidative addition step, observing that monocoordinated species (12e^−^) with electron‐rich and bulky ligands[^105^, ^106^, ^107^, ^108^, ^109^] are more active than the bidentate palladium complexes (14e^−^) that allow the OA of difficult substrates such as aryl chlorides. The OA of Pd(0) with electrophilic organohalides can follow two different reaction paths: SN2 or a three‐centre concerted mechanism,^[110]^ strongly depending on the solvent (see Scheme 11).^[111]^

In fact, the main feature of this step is the wide range of reagents and solvents that can be involved, giving rise to multiple mechanistic pathways,^[112]^ even though for monocoordinated palladium complex it is claimed that OA takes place via the three‐center concerted mechanism. In particular, it consists of a binding of the R^1^‐X molecule to the metal center and then a cleavage of the R^1^‐X bond forming the Pd‐R^1^ and Pd−X bonds simultaneously through a three‐center transition state. This mechanism leads to the retention of configuration at the stereogenic carbon atom in case of chiral electrophile R^1^‐X, and the relative *cis* disposition of ligands R^1^ and X after the oxidative addition. However, this *cis* conformation cannot be observed as the next *cis*‐to‐*trans* isomerization reaction of the oxidative product might be very fast, even though this is not demonstrated. On the other hand, monodentate complexes can give *trans* arrangement instead of *cis*, avoiding the isomerization step.

### 1.8 Transmetalation

**Scheme 12:**
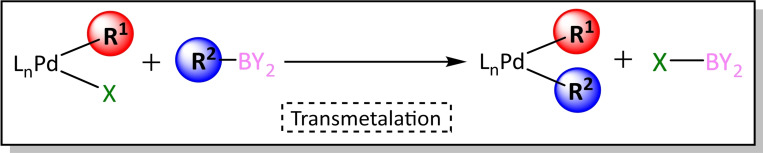
General equation for the transmetalation step.

Transmetalation is the reaction step where the organic group is transferred from one metal to another, and can be compared to an exchange of ligands between two metals. In addition, this step is the most characteristic one of C−C cross‐coupling reactions, as each cross‐coupling differs in the nucleophile used. In the case of the Suzuki‐Miyaura reaction (Scheme 12), the transmetalation step is an exchange between the organic group of the organoboron species (R^2^‐BY_2_) and the metal complex, in this case a Pd complex, with a halogen or pseudo‐halogen ligand (L_n_Pd^II^XR^1^).

**Scheme 13:**
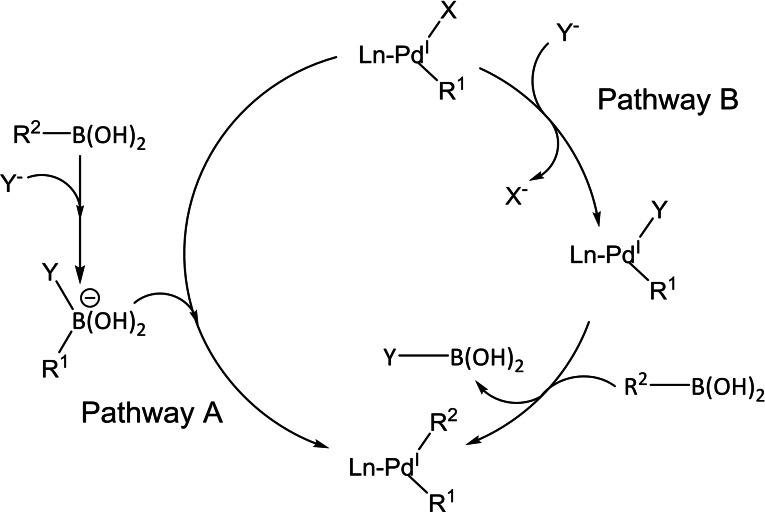
Plausible pathways of the transmetalation step: pathway A and pathway B.

Contrary to the first and last step of the SM catalytic cycle, the mechanim of transmetalation is not completely clear.^[113]^ Even though it is well known that the various organoboron species used participate actively in this step and a base is needed otherwise the reaction would not occur.^[114]^ Therefore, the requirement of a base^[61]^ leads to two hypothetical pathways (Scheme 13).^[36]^ In the first, *pathway A*, the base attacks the organoboron compound, forming an anionic *“ate complex”*, which is an organoboronate species, that will perform a nucleophilic attack on the palladium halide complex. Whereas the other mechanism, *pathway B*, occurs in two steps: first there is a ligand substitution between the halogen and the base, and then this new complex reacts with the neutral organoborane.

Pioneering computational studies done by Maseras et al.[^115^, ^116^, ^117^] in these two proposed pathways have shown that the pathway involving the anionic organoboronate species is the most plausible (*pathway A*). However, there has been some controversy in suggesting that the transmetalation follows the *pathway A*. The main disagreement comes from some kinetic studies done by Hartwig et al.,^[118]^ which show that under the SM typical conditions, using a weak base, the transmetalation follows the *pathway B* in which a palladium hydroxo complex and a neutral organoboron take part. Other experimental studies, made by Amatore, Jutand, and Le Duc[^119^, ^120^] are also in favour of the palladium hydroxo pathway because their kinetic studies show that in an excess of halide, the boronate pathway is inhibited, thus the palladium hydroxide pathway must be the correct one. On the other hand, recently, Lima et al.^[121]^ have reported an experimental analysis^[122]^ of the role of the base by performing some studies on the competition of two different boronic acids showing that the product distribution ratio depends on the nature of boronic acid, and the amount of the base added. This experimental study is in agreement with previous and new studies of Maseras and co‐workers[^123^, ^124^] in which the transmetalation step is the highest barrier thus the boronate pathway is faster than the palladium hydroxo route. However, it should be noted that even though transmetalation can occur through two different pathways, they both lead to the same intermediate, L_n_Pd‐R_1_R_2_, which has never been characterized.

### 1.9 Reductive elimination

**Scheme 14:**
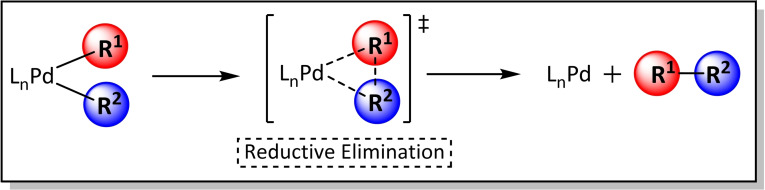
Mechanism for the reductive elimination step.

The reductive elimination is the last step in completing the catalytic cycle as well as being the reverse process of the oxidative addition. Thus, in this step, two bonds are cleaved and one bond, between two organic groups in *cis* position, is formed reducing the metal from Pd^II^ to Pd^0^, and the active catalyst is regenerated. More specifically, the oxidation state of the metal is reduced by two units. The reductive elimination is affected by different factors such as the characteristics of the ligands, like in the OA step.[^105^, ^125^, ^126^, ^127^] Recent studies on the effects of the ligands have shown that electron‐donating and bulky ligands favour this step, more concretely, the steric hindrance of the ligands reduce the lifetime of the transitory L_n_R^1^Pd^II^R^2^ species, thereby increase the rate of the reaction,[^120^, ^128^] and futher, adversely affects/prevents side reactions such as β‐*elimination*. Moreover, reductive elimination can occur through different mechanisms, although in monodentate Pd^II^ complexes the mechanism is *concerted* (Scheme 14). It is well known that this pathway involves the coupling of a single C−C bond via a three‐coordinated transition state.

### 1.10 New tendencies: amides the new key building blocks

**Scheme 15:**
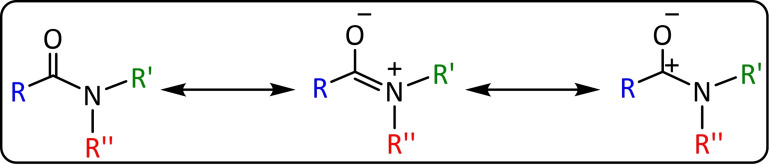
Resonance stabilization of amides.

Transition metal‐catalysed cross‐coupling is a powerful tool for assembling carbon‐carbon and carbon‐heteroatom bonds in a selective way. Although halides are the usual electrophilic partners, phenolic derivatives, also called “pseudohalides”, offer a valuable alternative since phenols are usually available and inexpensive materials. Recently, new ways of performing these assemblies have been reported by activating *C‐OR* bonds, using less reactive acyl electrophiles[^129^, ^130^, ^131^, ^132^, ^133^, ^134^, ^135^] such as esters, ethers, carbamates or sulfamates (Scheme 15). However, the use of amides as an electrophilic partner has become a challenge in the cross‐coupling field due to its high resonance stabilization,^[136]^ which causes low reactivity towards *C‐N activation* by the metal.^[137]^

Amides are known to be common functional groups that are key building blocks of proteins and are present in a wide range of other natural and unnatural compounds. Thus, utilization of this group in C−C or C−X bond formation would grant access to acyl‐metal intermediates from stable amide reagents and would provide a new C−C disconnection, increasing the scope of ketone synthesis. Although amides can be cleaved by enzymes such as proteases, breaking the C−N bond selectively using synthetic chemistry was impossible until 2015 when Szostak reported the study on N‐acyl‐glutarimides,^[138]^ which showed the relationship between twist angle and reactivity with the pioneering work on Ni catalyzed cross‐coupling reaction with amides, and also in 2015 Garg and co‐workers[^139^, ^140^, ^141^] reported the cross‐coupling reaction involving an amide; it was a SM coupling of amides catalysed by Ni, although the exact mechanism was unknown.

**Scheme 16:**
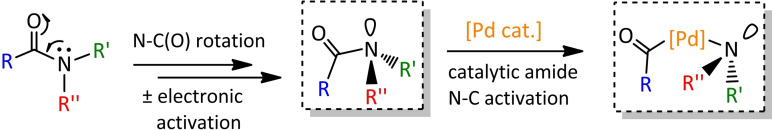
Proposed C−N activation of amides.

Following the pioneering research of Garg in cross‐coupling using amides, several investigations have focused on trying to understand the mechanism. Although it is well known that the huge gap in synthetic chemistry is due to the high activation energy required for the N−C(O) bond cleavage in amides, due to $n_{N}\to \pi_{C=O}{}^{*}\;$ conjugation, the Szostak's group^[142]^ has led to several breakthroughs in this field by proposing a generic method to activate the amide bond in transition metal catalysis.[^138^, ^143^, ^144^] This method is based on disrupting the conjugation by twisting the amide bond,^[145]^ approximately 50°, facilitating the metal insertion (Scheme 16).

**Figure 4:**
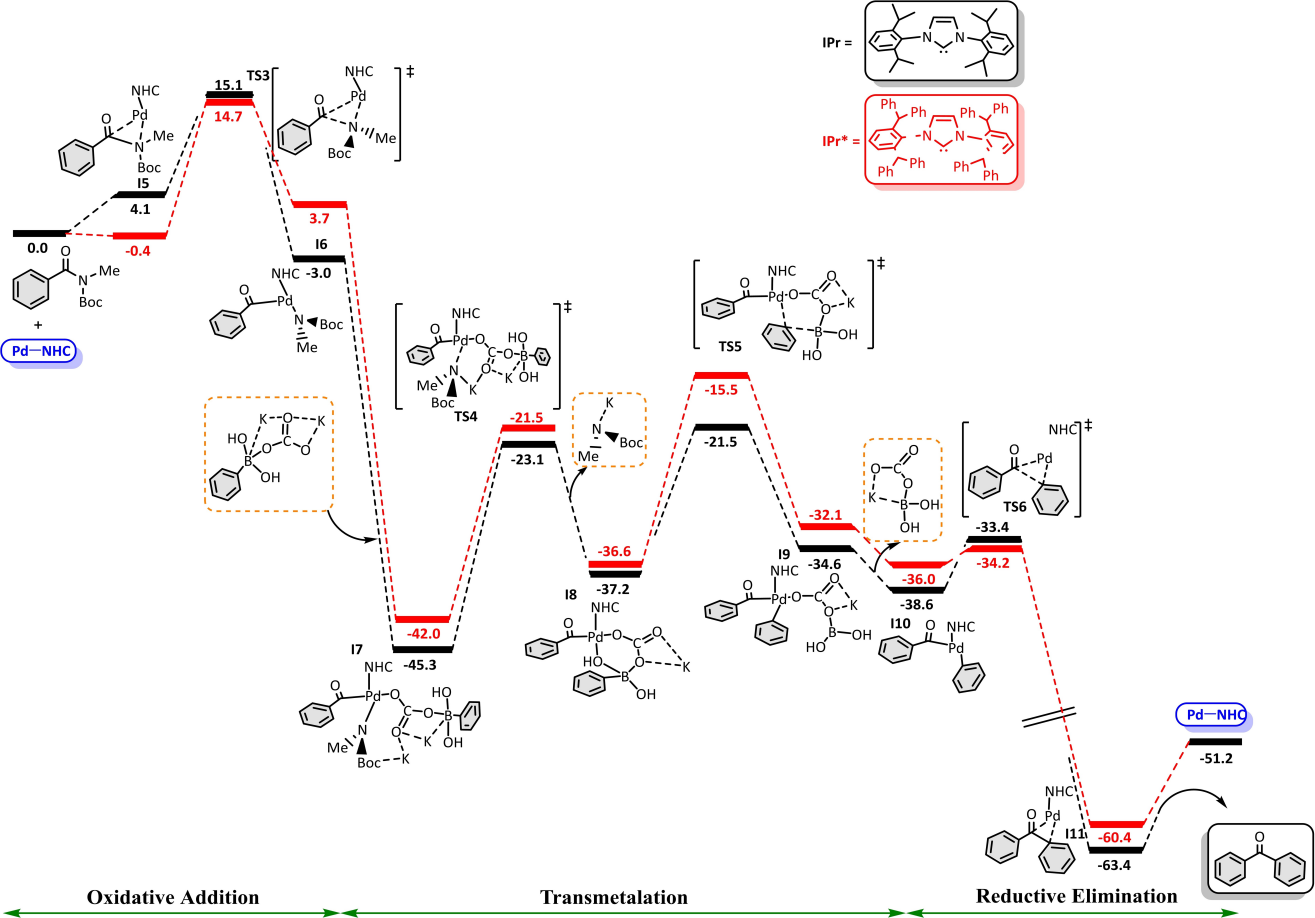
Free energy profile (in kcal/mol) of the overall catalytic cycle of the SMC of amides in THF as referred to Pd^0^(NHC), NHC=IPr (in black) and IPr* (in red). Figure extracted from Ref. [151].

Since a general method for activating the C−N was proposed,^[149]^ many Pd‐catalysed Suzuki‐Miyaura cross‐coupling reactions have been performed using amides with different substituents, particularly interesting in order to obtain biaryl ketones in high yields as they are important as motifs in pharmaceutical compounds.^[150]^ Following with the computational methods for the study of transition metal‐catalysed Suzuki‐Miyaura cross‐coupling reactions with amide substrates, the corresponding reaction mechanisms were better determined and/or understood for Pd‐NHC based catalysts, where NHC=*N,N*‐bis(2,6‐diisopropylphenyl)imidazol‐2‐ylidene (IPr) and *N,N* ‐bis(2,6‐bis(diphenylmethyl)‐4‐methylphenyl)imidazol‐2‐ylidene (IPr*). Here is a brief summary of the reasons that prompted Poater et al. to study these reactions and the particular goals achieved. Once, the experimental research groups of Nolan and Szostak demonstrated that the terminal substitution of the allyl scaffold in (NHC)‐Pd(allyl)Cl complexes results in a more facile activation of the complex to afford the catalytic species.[^137^, ^151^] DFT results provided the discussion not only of the precatalytic pathway for both NHC ligands, but mainly of the complete description of the Suzuki−Miyaura reaction for the catalytically active NHC−Pd(0) species using N‐acyl‐tert‐butyl‐carbamates and the boronic acid in the presence of K_2_CO_3_.^[94]^ The rate‐determining step of the catalytic process described in Figure 4,^[151]^ corresponding to the activation mechanism for the studied complexes is the formation of the C−O bond between the allyl and the carbonate moieties, and in the overall catalytic pathway it is the transmetalation. The exchange of IPr for IPr* slightly improves the catalysis, lowering the upper energy barrier, which confirms that the higher steric hindrance of IPr* helps somewhat the catalysis.^[152]^ %V_Bur_ indexes confirmed why this trend exists.[^153^, ^154^]

## 2 Conclusions

Knowing that catalysis is the way to escape stoichiometric reactions and at the same time to render reactions faster, the Suzuki‐Miyaura cross coupling reaction is generally catalyzed by taking palladium as a reference metal. In general, the rate determining step of the overall reaction pathway is not placed in the catalytic pathway but identified as one of the steps included in induction period. Thus, the generation of the catalytically active species is fundamental, and this leaves only selectivity for the catalytic cycle. Herein, is a description of the problem, emphasizing that in order to move from Pd(II) to Pd(0) one needs conditions that are anything but mild. Future synthetic, but also computational, efforts should lead to a better understanding of this reaction, and to enhance the catalytic activity by increasing the turnover number (TON).

To point out that there is a paucity of synthetic studies, in particular here in cross‐coupling reactions with Pd based catalysts, that the comparison of the reactivity of different precatalysts is not trivial. While it is understandable that the group that develops a given catalyst is typically focused on a single class of catalysts, it is a major impediment in synthetic applications that there are virtually no studies that compare various catalysts. This pertains to different Pd‐NHC catalysts and also to the comparisons between Pd‐NHCs and Pd‐phosphines.

Experimentally developed by Buchwald,[^155^, ^156^] Espinet,^[157]^ Mikami, Lassaletta,^[158]^ among others,^[159]^ the asymmetric version of the Suzuki‐Miyaura coupling is the next step to monitor by means of DFT calculations, however here the mechanistic insights were the main focus of the discussion.

## Conflict of interest

The authors declare no conflict of interest.

## Biographical Information

*Massimo Christian D'Alterio (Napoli, 1993) obtained his PhD in Chemistry in 2020 supervised by Dr. Giovanni Talarico, with a combined experimental/theoretical thesis on the mechanisms and stereoselectivity of ring opening polymerization reactions leading to eco‐friendly polymers. During his PhD he visited Dr. Albert Poater at the University of Girona, where he expanded his background of organometallic reactions; and Prof. Claudio Pellecchia at the University of Salerno, where he studied the synthesis and the characterization of polymers and copolymers of polylactic acid. Since 2020 he is a post‐doctoral researcher in the same group, where he applies his mixed theoretical and experimental know‐how for the optimization of catalytic reactions leading to environmentally benign polymers*.

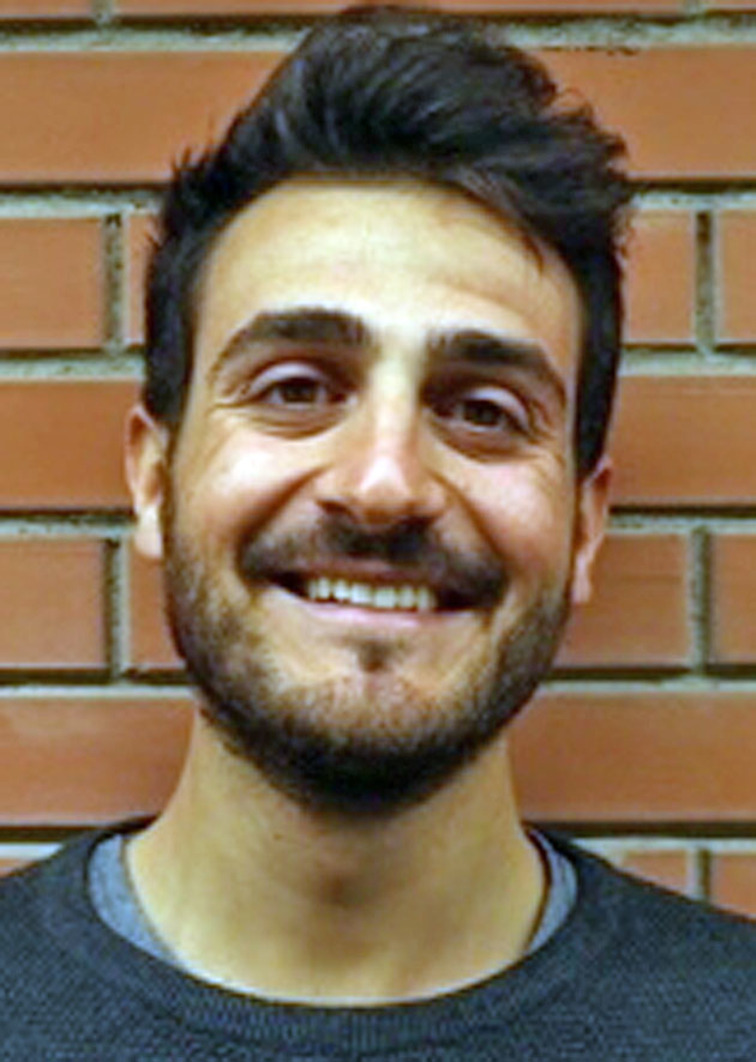

## Biographical Information

*Èric Casals‐Cruañas (Arenys de Mar, 1994) received his MSc in 2017 at University of Girona under the supervision of Dr. Albert Poater. His research was primarily focused on the reactivity of late transition metal N‐heterocyclic carbene complexes and their application in homogeneous catalysis by means of olefin metathesis and Suzuki‐Miyaura reactions. In 2019 he finished a MSc of CESIF. Since 2020 he works in the Regulatory Affairs Officer in ASPHALION*.

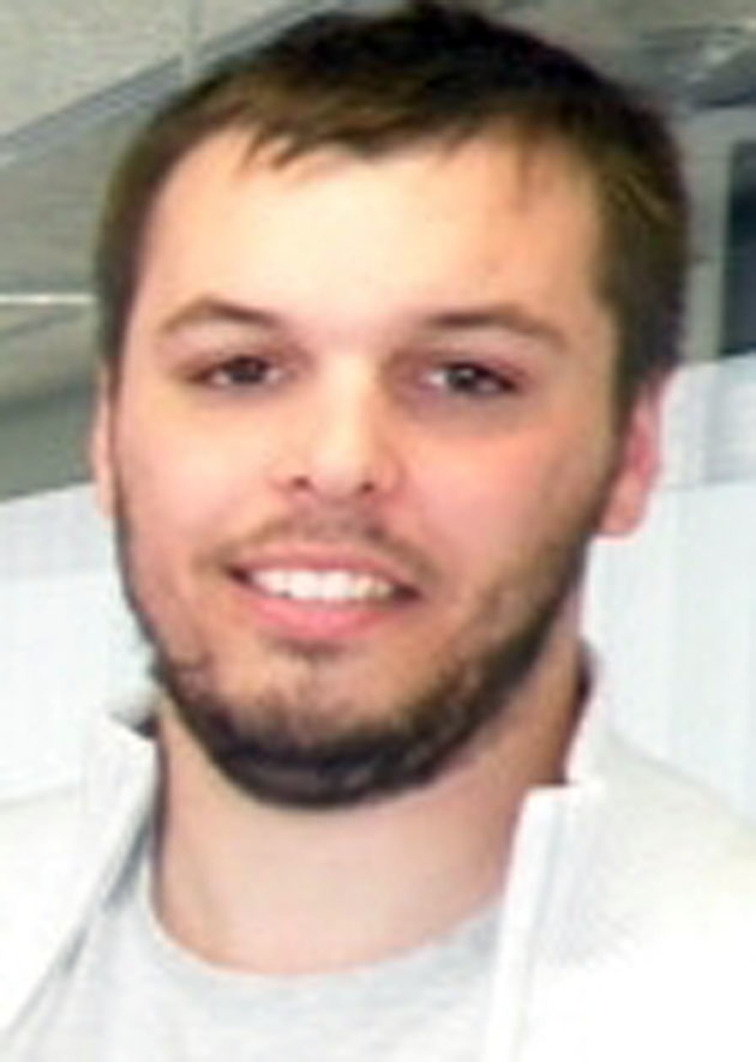

## Biographical Information

*Nikolaos Tzouras carried out his diploma thesis research in the university of Athens with Professor G. C. Vougioukalakis, on the synthesis of N‐Heterocyclic carbene precursors. After obtaining a scholarship for graduate studies in organic synthesis, he carried out his MSc research on sustainable metal catalyzed C‐H activation and multicomponent reactions, while he spent three months as a visiting researcher in Ghent university, working in the research group of Professor S. P. Nolan. Currently, he is pursuing a joint PhD degree with G. C. Vougioukalakis and S. P. Nolan as an FWO fellow, focusing on organometallic chemistry and catalysis*.

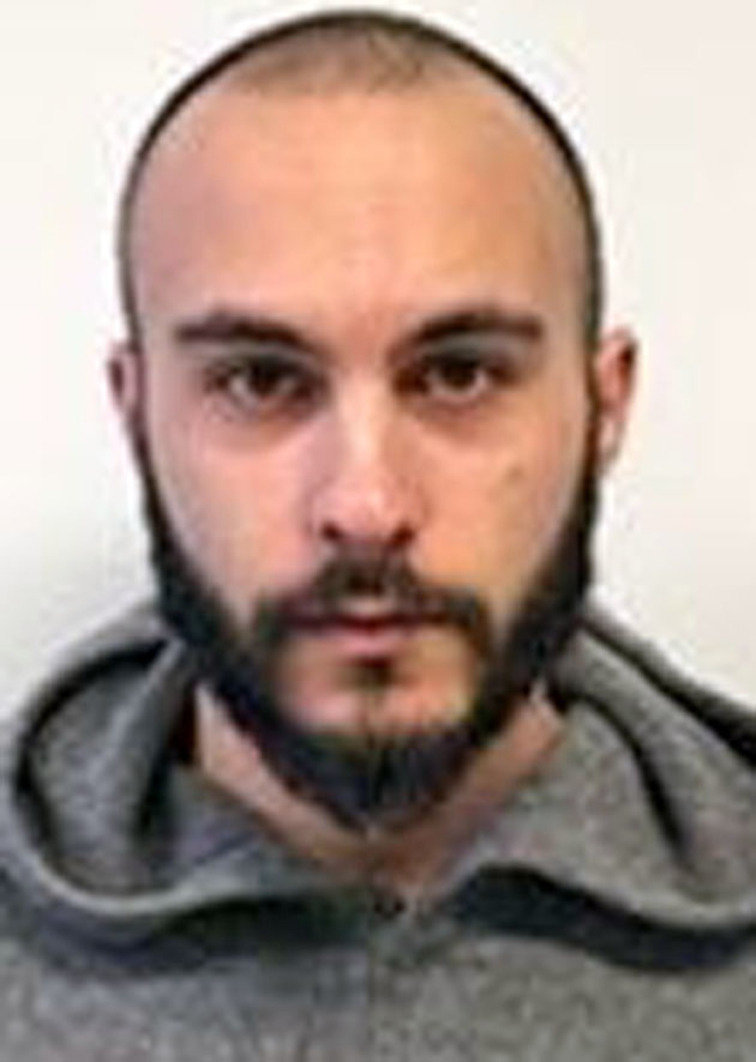

## Biographical Information

*Giovanni Talarico (Castellammare di Stabia, 1969) graduated in chemistry with honours in 1994 under the direction of Prof. Paolo Corradini. He received his PhD in the same University and subsequently completed a post‐doc in Katholieke Universiteit Nijmegen. He is Associate Professor at the Department of Chemical Sciences, University of Naples Federico II since 2006. In 2007 he was visiting professor at the CNRS in Strasbourg and since 2015 he has been collaborating with KAUST (Saudi Arabia). His main scientific interests concern the stereospecific metal catalyzed polymerization analyzed by experimental and theoretical analyzes and the structure‐property relationships of polymeric materials. He is the author of more than 100 papers*.

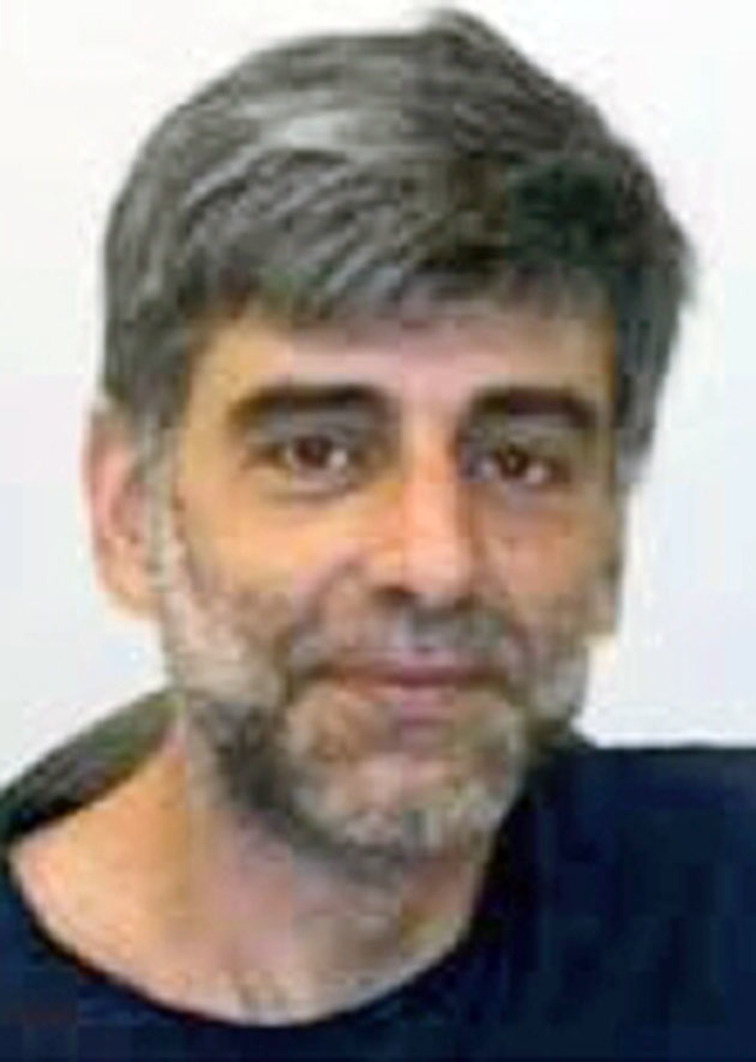

## Biographical Information

*Steven P. Nolan received his BSc in Chemistry from the University of West Florida and his PhD from the University of Miami where he worked under the supervision of Professor Carl D. Hoff. After a postdoctoral stay with Professor Tobin J. Marks at Northwestern University, he joined the Department of Chemistry of the University of New Orleans in 1990. In 2006 he joined the Institute of Chemical Research of Catalonia (ICIQ) as Group leader and ICREA Research Professor. In early 2009, he joined the School of Chemistry at the University of St Andrews and in 2017 joined the Department of Chemistry of Ghent University as Senior Full Professor. Professor Nolan's research interests revolve around the design and synthesis of catalytic complexes enabling organic transformations*.

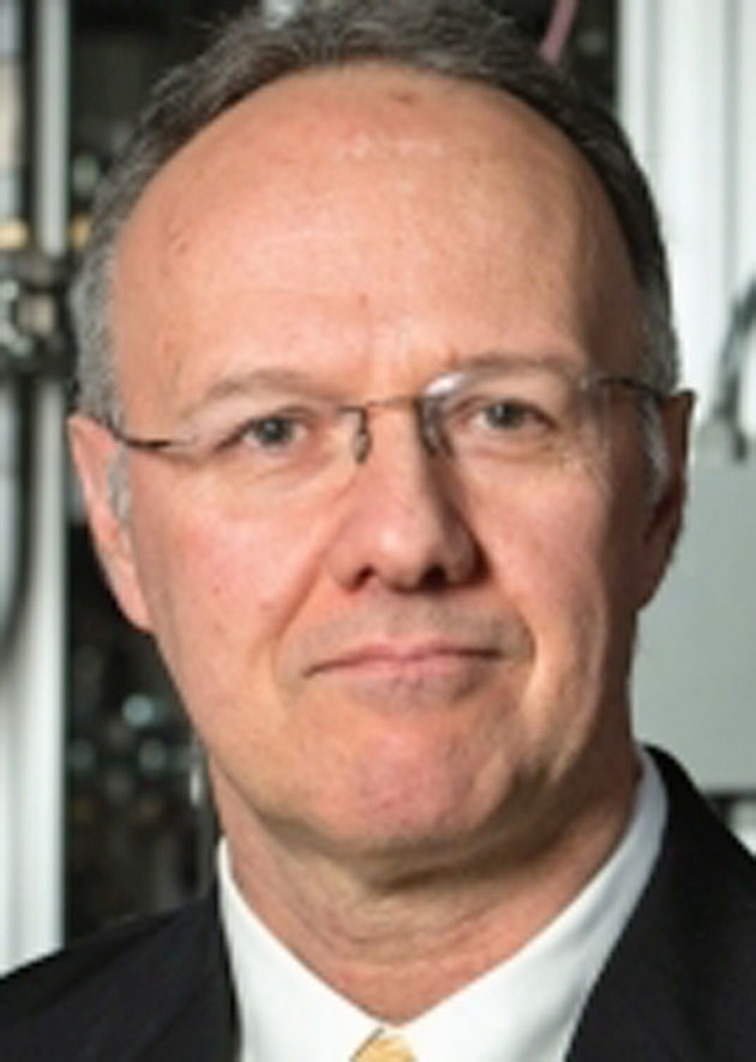

## Biographical Information

*Albert Poater (Banyoles, 1979) finished the PhD in Chemistry in 2006, supervised by Miquel Duran and Miquel Solà, at the University of Girona. After periods in Chile with Alejandro Toro‐Labbé; in Montpellier with Odile Eisenstein and a postdoc in University of Salerno with Luigi Cavallo, in 2010 he became an independent researcher as Ramón y Cajal and JIN in Girona, apart from Visiting Researcher at KAUST in Saudi Arabia, and with Remi Chauvin at LCC‐CNRS in Toulouse. As a whole, Dr. Poater has published over 200 papers, in DFT calculations on the mechanism of organometallic catalysis, mainly in green chemistry; and predictive catalysis by tools to describe the reactivity. In 2019 he became Associate Professor and received the ICREA Academia Award*.

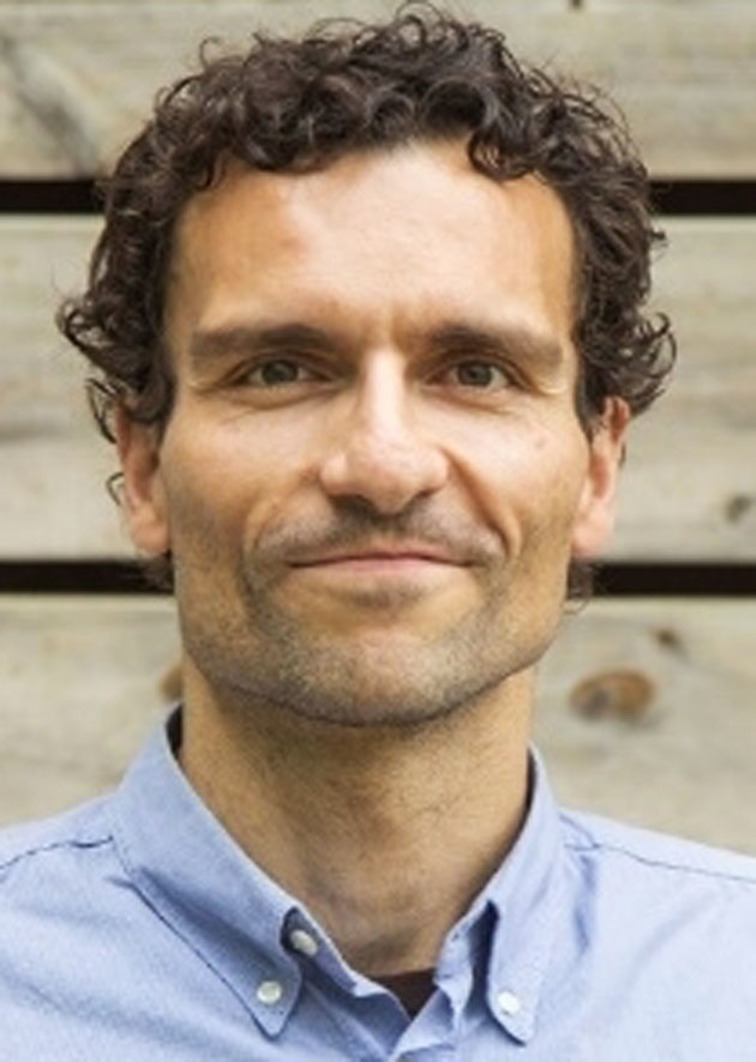

## Supporting information

As a service to our authors and readers, this journal provides supporting information supplied by the authors. Such materials are peer reviewed and may be re‐organized for online delivery, but are not copy‐edited or typeset. Technical support issues arising from supporting information (other than missing files) should be addressed to the authors.

Supporting Information
