# Supplementary material for: Mechanistic Aspects of the Palladium‐Catalyzed Suzuki‐Miyaura Cross‐Coupling Reaction
Source: Chemistry. 2021 Aug 9;27(54):13481–93. doi: 10.1002/chem.202101880 (PMC8518397; doi:10.1002/chem.202101880)
Supplement: Supplementary file 1 — Supporting Information [file CHEM-27-13481-s001.pdf]

Author Contributions

M.D. Writing – original draft:Equal
ÈricC.-C. Writing – original draft:Equal
N.T. Writing – original draft:Supporting; Writing – review & editing:Supporting
G.T. Writing – review & editing:Equal
S.N. Writing – review & editing:Lead
A.P. Writing – original draft:Equal; Writing – review & editing:Lead
